# Heart disease: recruitment of MEF2 activity by *β*−blockers wards off cardiomyocyte death

**DOI:** 10.1038/cddis.2015.293

**Published:** 2015-10-15

**Authors:** S Hashemi, S Wales, T Miyake, J C McDermott

**Affiliations:** 1 Department of Biology, York University, Toronto, Canada; 2 Muscle Health Research Centre (MHRC), York University, Toronto, Canada; 3 Centre for Research in Biomolecular Interactions (CRBI), York University, Toronto, Canada; 4 Centre for Research in Mass Spectrometry (CRMS), York University, Toronto, Canada

Morbidity and mortality associated with cardiovascular disease is a predominant global health problem. Death of cardiac muscle cells (cardiomyocytes) through programmed cell death (apoptosis) is one hallmark of the progression to heart failure.^1^ Moreover, loss of cardiomyocytes due to myocardial infarction (MI) in patients who survive the initial insult is a major determinant of residual heart function and ultimately their longer term prognosis. The extent of cardiomyocyte death is thus a primary determinant of subsequent left ventricular remodeling and progress to heart failure.^2^ Given that the human heart has virtually no innate capacity for regeneration, the mechanisms of cardiomyocyte cell death, and arguably more importantly, the survival of these cells and ultimately the salvage of the myocardium, is of profound clinical importance. An emergent concept in the report by Hashemi *et al.*^3^ is that a protein complex named MEF2, commonly known for its role in the development of the cardiovascular system, is linked to cardiomyocyte survival. In addition, the *β* adrenergic signaling pathway that fulfills an extensive role in the physiology and pathology of the adult heart is shown to intersect with MEF2's pro-survival function in cardiomyocytes.

The initial cue that led to the inception of these studies was accumulating evidence in the central nervous system indicating that a primary function of MEF2 in neurons is to protect them from apoptosis.^4, 5^ In view of the fundamental role played by MEF2 in the control of cardiac gene expression,^6^ it was a logical next step to address the question as to whether MEF2 might play a parallel role in the heart. Initially, using cultured rodent cardiomyocytes and flow cytometry as a sensitive indicator of apoptotic cell death, Hashemi *et al.*^3^ report that levels of cell death are dramatically enhanced in cardiomyocytes when the expression of MEF2 is suppressed using siRNA technology. Transcriptome analysis of these MEF2 depleted cells correspondingly indicated extensive differential expression in an apoptotic gene network suggesting therefore that MEF2 does indeed play a key role in cardiomyocyte survival.

A further clue to the potential importance of these observations came from previous work from the same group in which *β* adrenergic signaling mechanisms were found to directly converge on and regulate MEF2 activity.^7^ Acting through the *β* adrenergic receptors, catecholamines, such as adrenaline or noradrenaline, activate adenylate cyclase which, through the classical cAMP signaling pathway, result in activation of Protein kinase A (PKA). The striking connection was that the earlier work, in a skeletal muscle system, elucidated that PKA phosphorylates the MEF2 protein complex enhancing its interaction with a co-repressor (HDAC4), thus inactivating MEF2 function.^7, 8^ Underlying the relevance of these observations is the long known sensitivity of the heart to *β* adrenergic stimulation that exerts dynamic physiological control over both chronotropic (rate) and inotropic (strength) properties of cardiac contraction. Moreover, high levels of *β* adrenergic signaling, especially after MI, have been linked with cardiomyocyte death in the vicinity of the infarct and also in the more gradual loss seen in progressive heart failure.^1, 2^ What has perhaps not been fully appreciated is the connection of *β* adrenergic signaling to the acute and chronic control of myocardial gene expression and cell survival. Collectively, these observations framed the next question…Does *β* adrenergic signaling impact MEF2's pro-survival function in cardiomyocytes?

Experiments addressing this question indicate that treatment with agonists of the *β* adrenergic system acutely promotes cardiomyocyte apoptosis while concomitantly shutting down MEF2 function. A tantalizing additional layer of proof is that expression of an engineered form of MEF2 that is resistant to PKA in cardiomyocytes renders the cells less prone to apoptotic cell death in response to strong *β* adrenergic activation. Further evidence in this report are ‘proof of principle' experiments using *β* adrenergic blockers such as Atenolol, which competitively block the *β*1 adrenergic receptors from being activated by its natural ligands (Figure 1). To digress momentarily, since the Nobel prize winning development of this class of drugs by Sir James Black,^9^ *β*-blockers have truly proved to be a seminal ‘superdrug', used as a front line treatment for progressive heart disease and many other conditions for several decades. Enhanced sympathetic drive due to binding of catecholamines to the *β*1 and *β*2-subtypes of cardiac adrenergic receptors in patients with heart failure is inversely correlated with survival indicating one compelling reason why *β* blockers are efficacious in heart disease. In the studies of Hashemi *et al.*^3^ *β* blockers rescue MEF2 function from *β* adrenergic repression and, in so doing, promote cardiomyocyte survival (Figure 1). It is also pertinent, but as yet unanswered, to consider whether other types of cardiomyocyte death apart from apoptosis, such as autophagy-induced cell death and necroptosis, are also impacted by MEF2. Nevertheless, the logical extrapolation is to question whether *β* blockers can be effectively used to enhance MEF2 function and promote cardiomyocyte survival under conditions in which their survival is compromised, such as during acute and subacute phases after MI.

As with any basic discovery science, questions of efficacy and relevance to the human condition remain. A crucial one being, can timely *β* blocker treatment after MI and in the subacute stages in humans mitigate cardiomyocyte cell death, ventricular remodeling and progression to heart failure? Although there is no direct evidence addressing this question, one randomized trial reviewed by Sinert *et al.*^10^ demonstrated that *β* blocker treatment within 24 h in patients presenting with elevated ST segment MI did not reduce mortality or reinfarction when compared with placebo, indicating that *β* blockade post MI is tolerated with no obvious contraindications for most patients. The impact of this treatment modality for longer term cardiomyocyte survival remains to be determined. An alternate therapeutic approach might also be tailored drugs that transiently activate MEF2 through a different mechanism. No doubt there are myriad hurdles and complexities to consider before these observations can be clinically applied. Further experimentation in more physiological models and in human heart cells will examine the efficacy of these ideas. Moreover, detailed consideration of these findings requires further elaboration by clinical colleagues in the context of other treatment modalities and other cardiovascular system parameters.

## Figures and Tables

**Figure 1:**
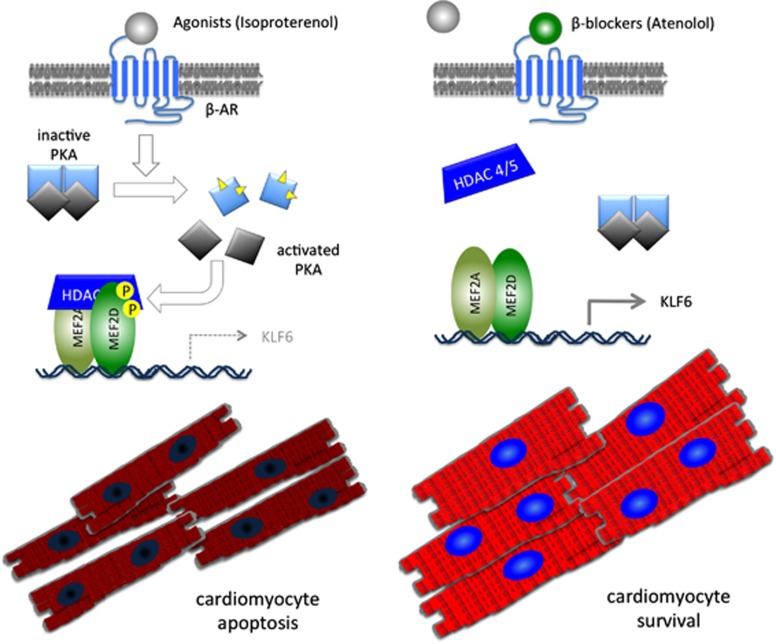
*β* adrenergic/PKA-mediated inhibition of MEF2 contributes to cardiomyocyte cell death. On the left side, acute activation of *β* adrenergic receptors invokes cAMP accumulation (yellow triangles) and PKA activation in cardiomyocytes, resulting in suppression of MEF2 transcriptional activity by direct phosphorylation and nuclear interaction with HDAC4/5. Expression of pro-survival genes such as *KLF6* is prevented resulting in enhanced cardiomyocyte death. On the right side, *β*-blockers, such as atenolol, competitively inhibit the activation of the *β* adrenergic receptors and downstream signaling resulting in enhanced MEF2 activity, thus promoting cardiomyocyte survival.
